# Identification of Usutu virus in blood donors as part of molecular screening for West Nile virus, France, 2023

**DOI:** 10.2807/1560-7917.ES.2026.31.43.2600044

**Published:** 2026-10-29

**Authors:** Pierre Gallian, Gilda Grard, Sophie Le Cam, Raphaelle Klitting, Marlène Guillet, Valérie Barlet, Marjorie Lacoste, Céline Ricard, Nadège Brisbarre, Christine Isnard, Laura Pezzi, Nazli Ayhan, Géraldine Piorkowski, Rayane Amaral, Thierry Peyrard, Marie Claire Paty, Florian Franke, Syria Laperche, Guillaume André Durand, Xavier de Lamballerie

**Affiliations:** 1 Etablissement Français du Sang, La Plaine Saint-Denis, Saint-Denis, France; 2 Unité des Virus Émergents (UVE: Aix-Marseille Univ-Corsica Univ-IRD 190-Inserm 1207-IRBA), Marseille, France; 3 National Reference Center for Arboviruses, National Institute of Health and Medical Research (Inserm) and French Armed Forces Biomedical Research Institute (IRBA), Marseille, France; 4 Etablissement Français du Sang Centre Pays de Loire, Nantes, France; 5 Etablissement Français du Sang Auvergne Rhône Alpes, Lyon Décines, France; 6 Etablissement Français du Sang Occitanie, Toulouse, France; 7 Etablissement Français du Sang Hauts de France-Normandie, Loos, France; 8 Santé publique France (French National Public Health Agency), Saint Maurice, France; 9 Santé publique France (French National Public Health Agency), Marseille, France

**Keywords:** Usutu virus, West Nile Virus, blood donors, NAT screening, France

## Abstract

**BACKGROUND:**

West Nile Virus (WNV) can be transmitted via blood transfusion and lead to neuroinvasive forms in recipients of blood products.

**AIM:**

We aimed to investigate WNV and Usutu virus (USUV) infections among blood donors in France.

**METHODS:**

Blood donations in 18 of 96 departments of metropolitan France and from travellers returning from affected areas were screened prospectively for WNV using a commercial nucleic acid test (NAT). In addition, stored samples from blood donations in the affected departments were retrospectively tested with RT-qPCR. Differentiation between WNV and USUV infections in NAT-positive samples was done with a RT-qPCR or by seroneutralisation after seroconversion.

**RESULTS:**

Between 18 July and 1 December 2023, 34 (0.018%) of 185,965 blood donation samples were initially positive for WNV. After investigations, 20 donors with USUV infections (16 confirmed, four probable) and five with WNV infections (three confirmed, two probable) were identified. In samples from four donors, WNV and USUV could not be differentiated, and five samples remained indeterminate. Seventeen USUV infections were considered autochthonous: nine departments in the southern and central parts of France, including two departments where WNV was also detected. Infections with USUV were detected between 21 July and 17 October and WNV between 23 August and 20 September.

**CONCLUSION:**

Via screening of blood donations, we could identify the circulation of USUV in 2023 in large parts of metropolitan France. A One Health approach is required to better understand the determinants of USUV circulation in France and the clinical consequences of USUV infections in humans.

**Key public health message**

**What did you want to address in this study and why?**

West Nile virus and Usutu virus can cause severe infections in humans and birds. These viruses are mainly transmitted via mosquitoes. After transfusion, cases of severe West Nile virus infection have been reported in blood recipients, while there are no reports for Usutu virus. We wanted to investigate West Nile virus and Usutu virus in blood donations in France.

**What have we learnt from this study?**

We tested almost 186,000 blood samples between July and December 2023 and detected Usutu virus in 20 samples and West Nile virus in five samples. Donors with Usutu virus were from southern and central France and those with West Nile virus from southern France. Usutu virus infections were detected between July and October and West Nile virus infections between August and September.

**What are the implications of your findings for public health?**

Usutu and West Nile virus infections were rare among blood donors in France. However, our results highlight the need to assess the risk and consequences of Usutu and West Nile virus infections via substances of human origin.

## Introduction

Usutu virus (USUV) and West Nile virus (WNV) are single-stranded RNA viruses belonging to the *Flaviviridae* family (species *Orthoflavivirus usutuense* and *Orthoflavivirus nilense,* genus *Orthoflavivirus*, subgenus *Euflavivirus*) within the Japanese encephalitis virus (JEV) serogroup.

Both viruses have been detected in Europe and Africa [1] and are considered to follow an enzootic cycle involving ornithophilic mosquitoes and migratory and resident birds as amplifying hosts and reservoirs. Humans and other mammals, including horses, are incidental dead-end hosts. Mass mortality of birds was reported in the United States (US) after the emergence of WNV in the Americas in 1999 and in Europe during the USUV epizootics in 2016 and 2018. The sustained circulation of WNV and USUV in Europe may result either from repeated reintroductions by migratory birds or from endemic transmission cycles maintained in resident bird populations [2,3].

In humans, most infections with WNV and USUV are asymptomatic or presenting with mild clinical symptoms. Therefore, infections in humans are often not diagnosed, except when an active surveillance of symptomatic cases is in place and when nucleic acid testing (NAT) for WNV as screening of blood donors is implemented. However, infections with WNV, and more rarely USUV, have been associated with neurological disorders, including meningitis and meningoencephalitis. The risk of severe and fatal outcomes is substantially higher for WNV than for USUV. Approximately 10% of patients with neuroinvasive WNV disease die. Severe forms of the disease are seen mainly in young children, older adults and people with weakened immune systems. Since 2019, three confirmed and one suspected fatal case have been reported among patients with weakened immune systems infected with USUV [4-7].

Since the emergence of WNV in North America, transmission of WNV via blood transfusion has been reported, with severe consequences for the recipients [8]. At least 76 confirmed or probable cases of transfusion-transmitted infections caused by 10 arboviruses have been described; WNV was involved in 42 of these [9]. In Europe, measures to prevent contamination by blood products have been implemented in accordance with recommendations of the European Centre for Disease Prevention and Control (ECDC) [10]. In 2012, two cases of WNV infection after blood transfusion were reported in Greece [11]. In European countries affected by WNV outbreaks, to ensure blood safety and to prevent the impact on self-sufficiency in blood products, particularly during the summer period, testing of blood donations with WNV-NAT in blood has been implemented despite cross-reactions with USUV.

In France, WNV infection has been a notifiable disease since 2021. Surveillance relies on case reporting to Santé publique France (the French National Public Health Agency), primarily laboratory confirmation of clinically suspected cases and molecular detection in asymptomatic blood donors. Infections with USUV are not mandatorily notifiable in France.

Here we describe detection of USUV in French blood donors, following the implementation of NAT screening for WNV during the 2023 season.

## Methods

### Nucleic Acid testing for West Nile virus in blood donations

#### Studied population

During the summer of 2023, NAT screening for WNV was implemented for all blood donations in 18 of the 96 departments of metropolitan France, following the detection of the first locally acquired human case in Gironde department on 28 July 2023. Seven departments were considered affected areas in which at least one probable or confirmed human case of autochthonous WNV infection had been reported in 2023: Alpes-Maritimes (D-06), Bouches-du-Rhône (D-13), Haute-Corse (D-2B), Charente (D-16), Charente-Maritime (D-17), Gironde (D-33) and Var (D-83). To monitor any potential geographical spread, six departments adjacent to these seven affected departments were selected by the French Public Transfusion Blood Service (Établissement Français du Sang (EFS)): Alpes-de-Haute-Provence (D-04), Hautes-Alpes (D-05), Corse-du-Sud (D-2A), Gard (D-30), Landes (D-40) and Vaucluse (D-84). Five departments, Côte-d’Or (D-21), Indre-et-Loire (D-37), Isère (D-38), Rhône (D-69) and Haute-Vienne (D-87), were included after detection of suspected WNV cases, later confirmed as USUV infections.

In addition, to avoid a 28-day deferral period, all blood donors returning from affected areas in France and elsewhere, including other countries, in accordance with the updated recommendations of the Higher Council for Public Health, were tested between 18 July and 1 December 2023 in all departments of metropolitan France.

The screening was carried out (i) in accordance with the French requirements on the selection of blood donors, which include, signing of a declaration of consent to the non-therapeutic use of blood products, and (ii) as part of the mandatory reporting of cases of WNV infection, which falls within the remit of Santé publique France.

After the detection of the first autochthonous human case of WNV infection in an affected area, earlier blood donations collected in the area and still available were quarantined and tested for WNV-RNA on archive samples. Due to the low available volume in these stored samples, RT-qPCR (confirmatory and discrimination methods) were used as test methods (see below).

#### Screening assay

Routine EFS laboratories tested each donor sample individually with WNV Nucleic Acid Test, using the Cobas WNV assay on the Cobas 8800 automate (Roche, Basel, Switzerland). This assay is intended to detect RNA of WNV but allows genomic detection of related Orthoflaviviruses by cross-reactivity.

Positive samples (as defined by the manufacturer) were systematically retested in duplicate using the same assay and classified as repeatedly (at least one positive among the duplicates) or non-repeatedly positive (both replicates negative) samples. Persons with positive test results were considered as potential WNV cases and were notified to public health authorities, which implies contacting and informing the blood donors concerned. This reporting is based on the official French public health questionnaire and includes sections on results of the diagnostic tests, description of clinical symptoms and travel history.

#### Molecular testing for confirmation and discrimination

Confirmatory testing was performed at the National Reference Center for Arboviruses using in-house dual target RT-qPCR systems from 165 µL of plasma on the Panther Fusion automated system (Hologic, San Diego, US). The two amplification systems used in a duplex test were performed as described for testing for WNV [12,13] and USUV [14,15]. An internal extraction and amplification control was used in both tests. The limits of detection (LOD95) were 3 RNA copies/µL plasma for WNV and 1 RNA copies/µL plasma for USUV. In case of negative results, a new extraction was performed from a larger volume of plasma (600 µL if available) and tested on a CFX thermocycler (Bio-Rad, Hercules, US), using the WNV-specific RealStar RT-PCR kit (Altona Diagnostics GmbH, Altona, Germany) and the aforementioned USUV dual target test.

### Genotyping

For genotyping of USUV, sequences from samples positive in RT-qPCR were obtained using the Ion Torrent sequencing technology (S5 sequencer) after a pre-amplification step with a virus-specific primer set allowing to generate 35 amplicons of 400–500 bp spanning the entire genome of USUV [16]. Sequences were aligned using multiple alignment using fast Fourier transform (MAFFT) version 7.511 (https://mafft.cbrc.jp/alignment/server/index.html) with a set of reference sequences representative of USUV genotypes. Alignments were then inspected manually with AliView version 1.0 (https://ormbunkar.se/aliview/downloads), and the alignment was trimmed to the resolved part of each partial sequence (nt positions 94–1002 of the coding sequence (CDS) of USUV for BD-01 and positions 2338–3826 of the CDS for BD-02). Phylogenetic relationships were inferred using a maximum likelihood (ML) approach with IQ-Tree version 1.6.12 (https://iqtree.github.io), using the best-fit model identified by ModelFinder (Tne + I and Tne + G4 for samples BD-01 and BD-02, respectively) and assessed branch support using an ultrafast bootstrap approximation (UFBoot2) (1,000 replicates). Ambiguous positions were not masked. Sequence and analysis files are available from https://github.com/rklitting/WNV_USUV_EFS_2023.

### Serological testing

Presence of antibodies was investigated especially in samples that could not be differentiated by molecular assays. To identify a seroconversion profile, a late (> 4 weeks) sample was requested. Antibodies (WNV: IgM and IgG and USUV: IgG) were tested by the National Reference Center for Arboviruses using commercial ELISA assays (Euroimmun, Lübeck, Germany).

#### Neutralising activity of anti-WNV and anti-USUV antibodies

Neutralising antibodies were detected using a virus neutralisation test (VNT) as previously described [17]. We used Vero E6 cells cultured in 96-well microplates, 100 TCID50 (50% tissue culture infectious dose) of the WNV strain (R94224 isolated in 2008 in the US) or USUV strain (isolated in Senegal in 2010) and serial dilutions of serum (1/20–1/160). Dilutions associated with cytopathic effect due to viral growth (CPE) and no-CPE (neutralisation of virus growth by specific antibodies) on day 4 post-infection were considered as negative (no neutralisation) and positive (complete neutralisation), respectively. The neutralisation titre referred to the highest positive dilution of serum. Specimens with a titre ≥ 20 were considered positive. All samples were tested twice for all dilutions.

### Classification of cases

Cases were classified as confirmed, probable, undifferentiated (WNV/USUV) and indeterminate case for WNV or USUV infection (Box). The classification was primarily based on molecular results and, when necessary, on serological results from the first donation and, when available, from a sample collected 4–6 weeks after the first donation. Criteria differed slightly between the prospective (based on the routine primary WNV-NAT-screening assay) and retrospective (based on archive samples) studies.

Box **Definitions of cases with West Nile virus or Usutu virus, France, 2023**

**Confirmed case with WNV or USUV infection:**

- Prospective study: detection of WNV or USUV with a species-specific RT-qPCR
- Retrospective study: detection of WNV or USUV with a species-specific RT-qPCR with Cq < 40
  OR
- Detection of neutralising antibodies with titre ≥ 80 and a difference ≥ 2 dilutions between both viruses

**Probable case with WNV or USUV infection:**

- Detection of anti-WNV IgM in the first donation and IgG seroconversion in a later sample
  OR
- Fourfold increase of IgG titre in a later sample
  OR
- Detection of neutralising antibodies with a titre ≥ 40 and a difference ≥ 2 dilutions between both viruses

**WNV or USUV infection undifferentiated:**

- Detection with a non-species-specific RT-PCR and species-specific RT-qPCR negative
- Detection of anti-WNV IgM in first donation and not detected in a later sample
- Detection of neutralising antibodies without discrimination between WNV and USUV

**Indeterminate:**

- Prospective study: samples tested positive by WNV-NAT and not classified as confirmed or probable or undifferentiated
- Retrospective study: detection of WNV or USUV with a species-specific RT-qPCR with Cq ≥ 40 OR no seroconversion

Cq: quantitative cycle; USUV: Usutu virus: WNV: West Nile virus.

## Results

Between 18 July and 1 December 2023, 185,180 blood donations were analysed with WNV-NAT, including donations collected in 18 of 96 departments in metropolitan France and in travellers returning from affected areas. In addition, 785 archived samples were retrospectively tested with RT-qPCR. Of these 185,965 samples, 34 (0.018%) tested positive and were further investigated: 29 from the prospective screening (26 repeatedly positive and three non-repeatedly after several testing iterations) and five from the retrospective screening (Figure 1). These five samples could not be retested because of a low sample volume. After further tests, 19 cases were considered confirmed: 16 with USUV (RT-qPCR: n = 11; serology: n = 5) and three with WNV (RT-qPCR: n = 2; serology: n = 1) infection. Six cases were probable based on serology (USUV: n = 4; WNV: n = 2). In four repeatedly positive samples, USUV or WNV infection could not be distinguished, and these were categorised as WNV/USUV undifferentiated. Five samples were considered as indeterminate.

**Figure 1:**
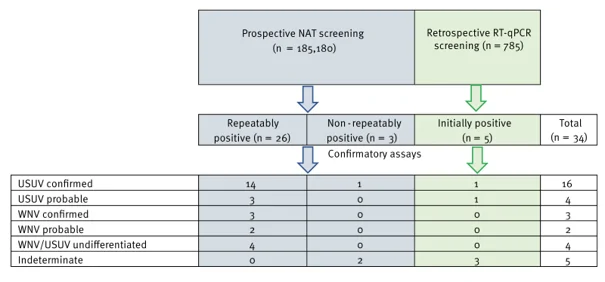
Flowchart of detection of Usutu and West Nile virus in samples from blood donations, France, 18 July–1 December 2023 (n = 185,965)

NAT: nucleic acid testing; USUV: Usutu virus; WNV: West Nile virus.

Confirmed case: Detection of WNV or USUV with a species-specific RT-qPCR or detection of neutralising antibodies with titre ≥ 80 and a difference ≥ 2 dilutions between both viruses.

Probable case: Detection of anti-WNV IgM in the first donation and IgG seroconversion in a later sample or fourfold increase of IgG titre in a later sample or detection of neutralising antibodies with titre ≥ 40 and a difference ≥ 2 dilutions between both viruses.

### Confirmed and probable cases

In the prospective screening, USUV infections were detected between 21 July (week 29) and 17 October (week 42) and WNV infections between 23 August (week 34) and 20 September (week 38) (Figure 2).

**Figure 2:**
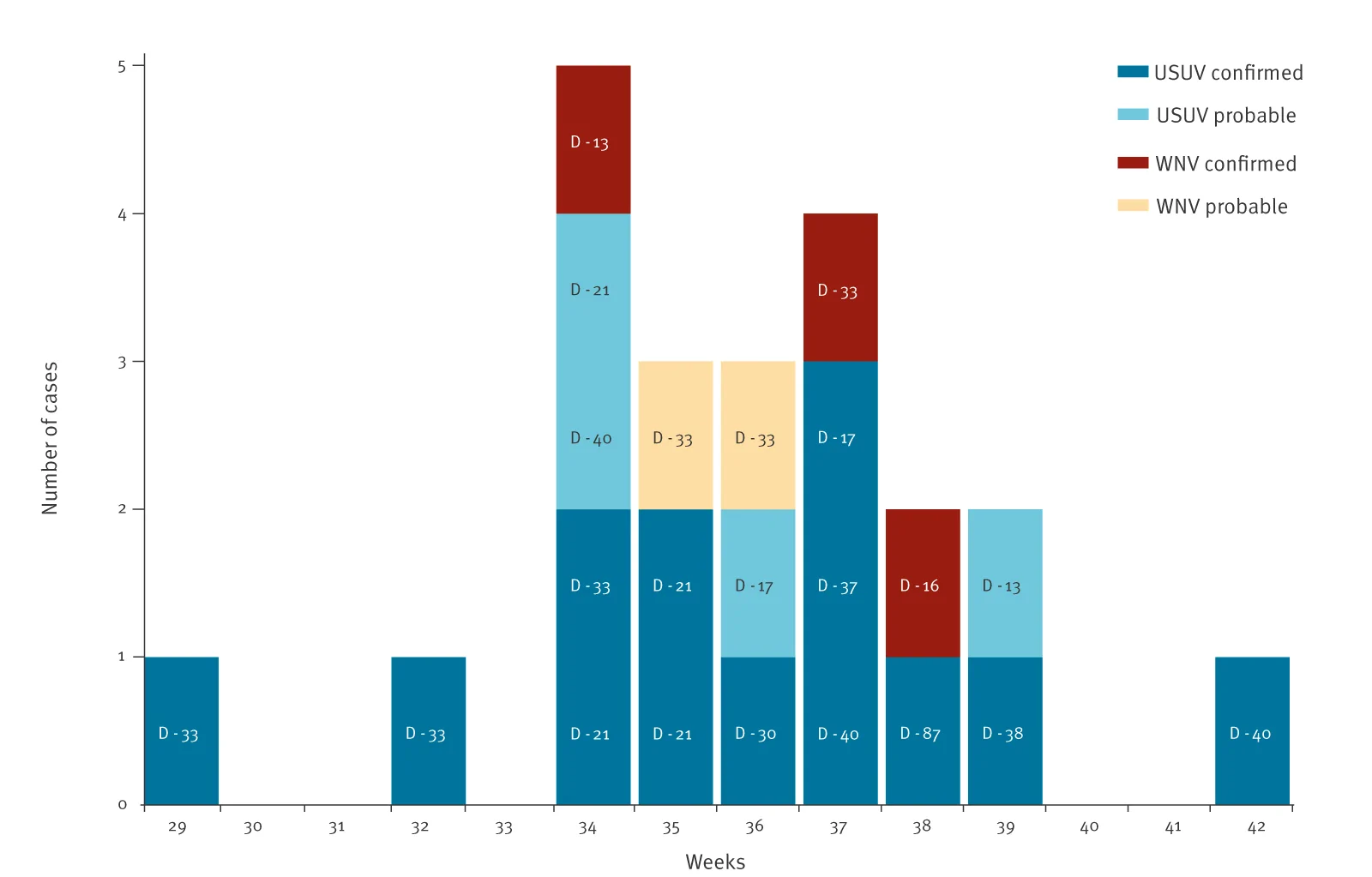
Timeline of detection of confirmed and probable cases of autochthonous Usutu virus and West Nile virus infections in blood donations, France, 18 July–1 December 2023 (n = 22)

D-13: Bouches du Rhône; D-16: Charente; D-17: Charente-Maritime; D-21: Côte d’Or; D-33: Gironde; D-37: Indre et Loire; D-38: Isère; D-40: Landes; D-87: Haute Vienne; USUV: Usutu virus; WNV: West Nile virus.

Confirmed case: Detection of WNV or USUV with a species-specific RT-qPCR or detection of neutralising antibodies with titre ≥ 80 and a difference ≥ 2 dilutions between both viruses.

Probable case: Detection of anti-WNV IgM in the first donation and IgG seroconversion in a later sample or fourfold increase of IgG titre in a later sample or detection of neutralising antibodies with titre ≥ 40 and a difference ≥ 2 dilutions between both viruses.

Of the 34 blood donors initially tested positive, 22 were males and 12 were females. Twelve of the 20 confirmed or probable cases with USUV infection were males, eight were females. Four of the five confirmed or probable cases with WNV infection were males and one was female.

All 34 blood donors were asymptomatic at the time of donation and were further interviewed for the occurrence of clinical signs 14 days before or after the donation. Among the 25 confirmed and probable cases, six donors reported mild clinical symptoms (Table 1).

**Table 1:** Symptoms of confirmed and probable cases of Usutu virus and West Nile virus infection among blood donors tested prospectively and retrospectively, France, 18 July–1 December 2023 (n = 25)^a^

Case category	Symptomatic (n)	Time between symptoms and donation	Symptoms
Usutu virus infection (n = 20)			
Confirmed (n = 16)	3	14 days before	Asthenia, myalgia, arthralgia, digestive disorders
		4 days before	Retro-orbital pain
		7 days after	Skin rash
Probable (n = 4)	2	5 days before	Mild diarrhoea without fever
		5 days after	Mild fever, asthenia
West Nile virus infection (n = 5)			
Confirmed (n = 3)	1	4 days before	Asthenia, low back pain, headache
Probable (n = 2)	0	Not applicable	

^a^ Self-reporting of symptoms to an interviewing physician.

Confirmed case: Detection of WNV or USUV with a species-specific RT-qPCR or detection of neutralising antibodies with titre ≥ 80 and a difference ≥ 2 dilutions between both viruses.

Probable case: Detection of anti-WNV IgM in the first donation and IgG seroconversion in a later sample or fourfold increase of IgG titre in a later sample or detection of neutralising antibodies with titre ≥ 40 and a difference ≥ 2 dilutions between both viruses.

### Geographical distribution

Three of the 20 cases with confirmed or probable USUV infection were probably infected abroad (Italy, Spain and Türkiye/Central Europe) based on the travel history reported during the pre-donation interview and a consistent incubation period. Seventeen (13 confirmed and four probable) were considered autochthonous cases, and they resided in nine departments (Figure 3, Table 2). In departments where USUV infections were detected (Figure 3), the mean detection rate for positive USUV-RNA was 2.0/10,000 donations, ranging from 0.6 to 5.3/10,000 donations in the Bouches du Rhône (D-13) and in the Landes (D-40) departments, respectively (Table 2). These estimates should be interpreted with caution due to the heterogeneity of the screening areas (varying population density and rural vs urban), as well as the uncertainty about the timing of screening in relation to the viral circulation—all factors that may influence the ability to detect USUV in blood donors. The five confirmed or probable WNV cases were considered locally acquired and resided in three departments (Bouches du Rhône (D-13), Charente (D-16) and Gironde (D-33). Co-circulation of WNV and USUV was observed in two departments (Bouches du Rhône (D-13) and Gironde (D-33) (Figure 2, Figure 3).

**Figure 3:**
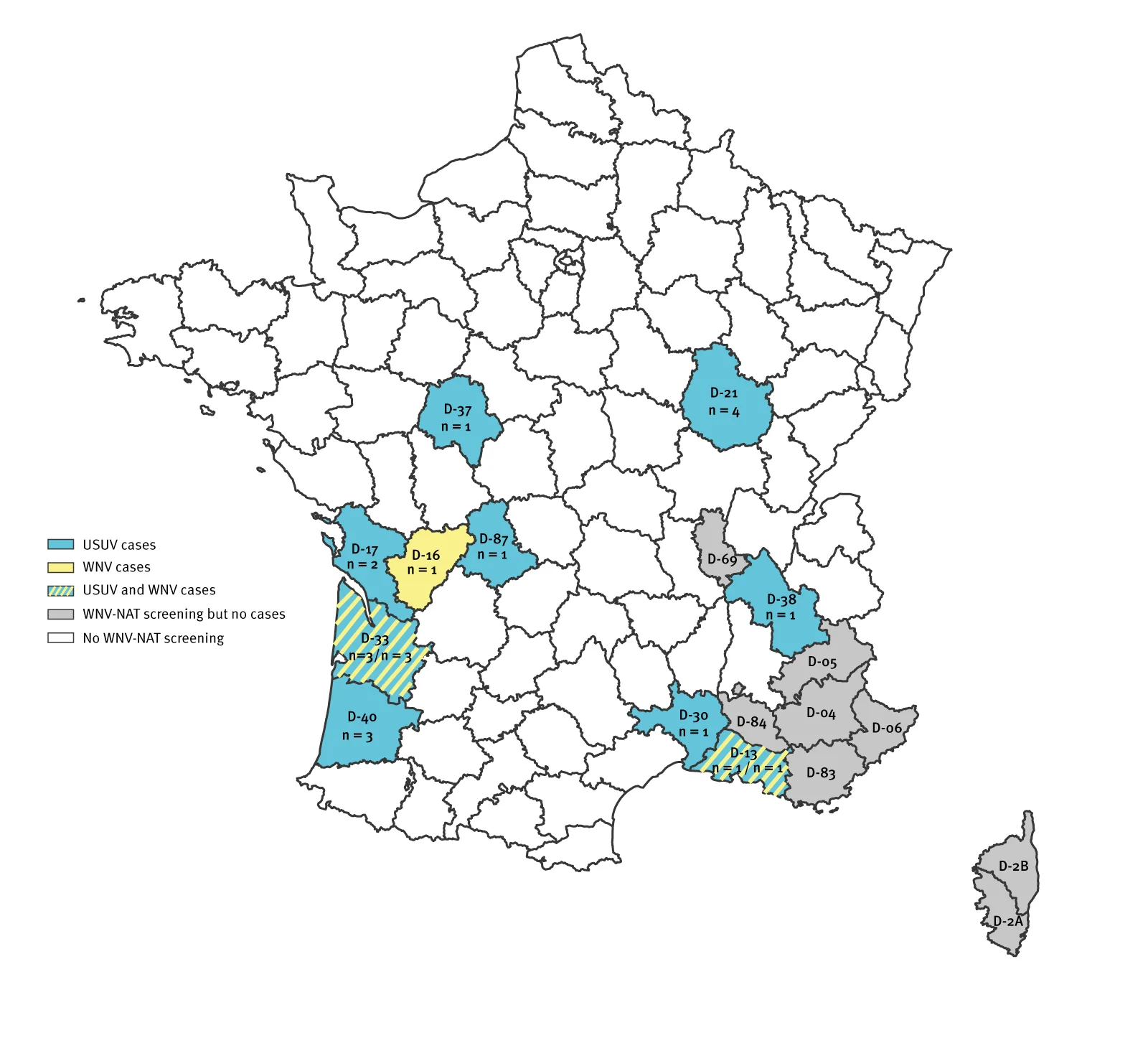
Geographical distribution of confirmed and probable autochthonous cases of Usutu (n = 17) and West Nile (n = 5) virus infection among blood donors, France, 18 July–1 December 2023

D-2A: Corse-du-Sud; D-2B: Haute-Corse; D-04: Alpes-de-Haute-Provence; D-05: Hautes-Alpes; D-06: Alpes-Maritimes; D-13: Bouches du Rhône; D-16: Charente; D-17: Charente-Maritime; D-21: Côte d’Or; D-30: Gard; D-33: Gironde; D-37: Indre et Loire; D-38: Isère; D-40: Landes; D-69: Rhône; D-83: Var; D-84: Vaucluse; D-87: Haute Vienne; NAT: nucleic acid test; USUV: Usutu virus; WNV: West Nile virus.

Confirmed case: Detection of WNV or USUV with a species-specific RT-qPCR or detection of neutralising antibodies with titre ≥ 80 and a difference ≥ 2 dilutions between both viruses.

Probable case: Detection of anti-WNV IgM in the first donation and IgG seroconversion in a later sample or fourfold increase of IgG titre in a later sample or detection of neutralising antibodies with titre ≥ 40 and a difference ≥ 2 dilutions between both viruses.

The number of cases in the department is marked below the department code.

**Table 2:** Timeframe for molecular screening of blood donors for West Nile virus and detection of Usutu virus, by department, France, 18 July–1 December 2023 (n = 185,965)

Department	Samples tested (n)	USUV RNA detected		Start of testing
		n	‱	
Alpes de Haute Provence (D-04)	1,057	0	0	31 Aug
Hautes-Alpes (D-05)	784	0	0	31 Aug
Alpes-Maritimes (D-06)	4,846	0	0	17 Aug
Bouches du Rhône (D-13)	16,639	1	0.60	17 Aug
Charente (D-16)	4,596	0	0	17 Aug
Charente-Maritime (D-17)	9,887	2	2.02	10 Aug
Corse du Sud (D-2A)	1,336	0	0	30 Aug
Haute-Corse (D-2B)	1,226	0	0	30 Aug
Côte d’Or (D-21)	8,085	4	5.00	5 Sep
Gard (D-30)	5,398	1	1.85	31 Aug
Gironde (D-33)	20,019	3	1.50	28 Jul
Indre et Loire (D-37)	5,529	1	1.81	20 Sep
Isère (D-38)	10,410	1	0.96	3 Oct
Landes (D-40)	5,661	3	5.30	17 Aug
Rhône (D-69)	13,042	0	0	25 Sep
Var (D-83)	7,133	0	0	12 Aug
Vaucluse (D-84)	2,980	0	0	29 Aug
Haute Vienne (D-87)	3,808	1	2.63	30 Sep
Other departments (travellers)	63,529	3	0.47	18 Jul

USUV: Usutu virus.

Confirmed case: Detection of USUV with a species-specific RT-qPCR or detection of neutralising antibodies with titre ≥ 80 and a difference ≥ 2 dilutions between both viruses.

Probable case: Detection of anti-WNV IgM in the first donation and IgG seroconversion in a later sample or fourfold increase of IgG titre in a later sample or detection of neutralising antibodies with titre ≥ 40 and a difference ≥ 2 dilutions between both viruses.

### Genomic sequencing and typing of viral strains

Due to a low viral load in the samples, we could obtain only two USUV partial sequences (genome covered at 10X: 7.3% and 13.5%, respectively). For sample BD-01, nucleotide positions 94–1002 of the CDS of USUV were covered, and for sample DB-02, positions 2338–3826 of the CDS were covered. By aligning each partial sequence with virus genomes representative of USUV genotypes and inferring their phylogenetic relationships, we found that both partial sequences most likely belonged (bootstrap support > 95) to the Africa 3 genotype (Figure 4), aligning with what has been most frequently encountered in France in recent years.

**Figure 4:**
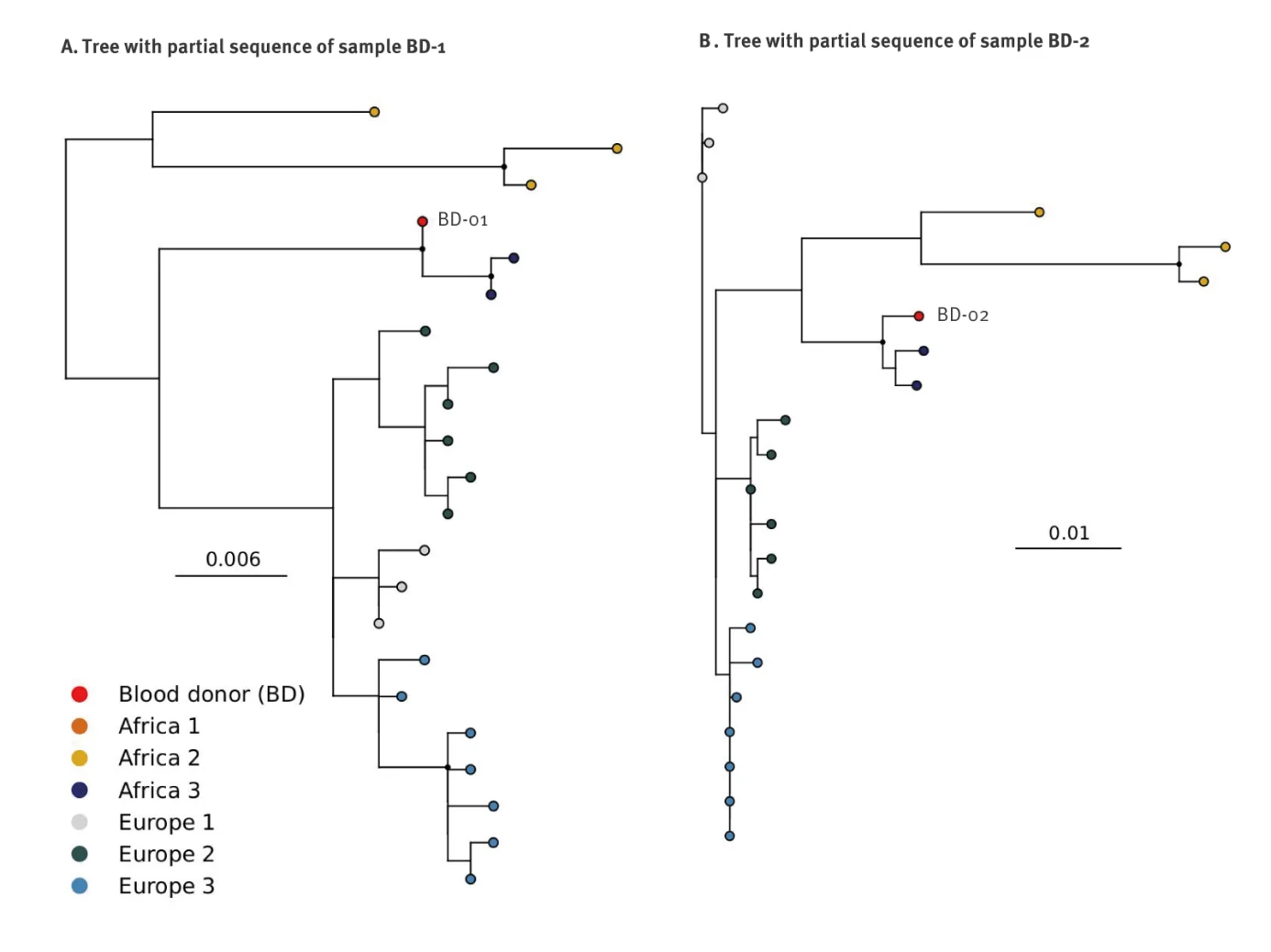
Phylogenetic assignment of genotypes for two Usutu virus partial sequences, France, 2023

For samples BD-1 (Panel A) and BD-2 (Panel B), the partial sequence was aligned with sequences representative of USUV genotypes (KC754958 for Africa 1; KX601692, KF573410 and AY453412 for Africa 2; KY426765 and KY128482 for Africa 3; JQ219843, EF206350 and AY453411 for Europe 1; JF266698, MF063042, MF063043, HM569263, KX555628 and KX555627 for Europe 2; KY128481, KJ438769, KX977447, KX601690, KX601691, KJ859682, and KJ859683 for Europe 3). The alignment was trimmed to the resolved part of each partial sequence (nt positions 94 to 1,002 of the coding sequence (CDS) of USUV for BD-1 and positions 2338 to 3826 of the CDS for BD-2) and a phylogeny was inferred using a maximum likelihood approach with IQ-Tree (version 1.6.12), with the best-fit model identified by ModelFinder (Tne + I and Tne + G4 for BD-1 and BD-2, respectively) and branch support was assessed using an ultrafast bootstrap approximation (UFBoot2) (1,000 replicates). Ambiguous positions were not masked. The tree was rooted using the Africa 1 genotype of USUV. Tips corresponding to sequences from blood donors are highlighted by red circles. Nodes with a bootstrap support above 95 are shown by a black circle.

## Discussion

Usutu virus was identified in 1959 in South Africa. In Europe, it was first reported in 1996 in Italy (Tuscany region) in a retrospective study [15] and the first fatal cases in the avian population linked to the virus were reported in 2001 in Austria [18]. Subsequently, USUV has been detected in birds, mosquitoes and horses in many European countries: Belgium, Croatia, Czechia, Germany, Greece, Hungary, Italy, the Netherlands, Poland, Serbia, Slovakia, Spain, Switzerland and the United Kingdom (UK) [19]. In France, USUV infections in birds have been reported regularly since 2015 [20]. In 2018, mass deaths of wild birds were observed (103 birds tested positive) in 46 departments in metropolitan France [21].

The first human cases of USUV infection in Europe, in 2009, were two Italian patients presenting with neurological symptoms [22,23]. Between 2009 and 2021, 115 cases of acute USUV infection were reported in Europe, including 20 with neuroinvasive forms (meningitis or meningoencephalitis) and one with idiopathic facial paralysis [24]. Since then, USUV has been detected in blood donations in several countries (Austria: n = 27 [25]; Germany: n = 10 [26]; Spain: n = 3 [27]; Italy: n = 1 [28]) and in patients, some of whom presented with neurological forms (Croatia: n = 5 [29], Czechia: n = 1 [30]), including one case reported in an immunocompetent patient [31]. Neurological forms have been mainly seen in patients with various types of comorbidities. In France, the first symptomatic human cases were recorded in 2016 in the Hérault department (D-34, Mediterranean coast) [32] and a febrile form in 2022 in the Landes department (D-40, Atlantic coast).

Via WNV-NAT screening of blood donations, we were able to detect the circulation of USUV in nine departments of the country. We identified 17 autochthonous and three imported cases of Usutu virus among the 34 donors with initially WNV-NAT positive samples. West Nile virus NAT screening assays used in Europe for blood transfusions are highly sensitive but cross-react with viruses phylogenetically related to WNV, in particular USUV and Japanese encephalitis virus [33,34]. Between 2016 and 2024, more than 100 WNV-NAT positive and asymptomatic blood donors, who were subsequently found USUV-RNA positive, were identified; most of them in Austria and Italy [25,35-37], but also in Germany [26,38], Spain [27] and the Netherlands [39]. A summary of these cases is provided in Supplementary Table S1. It is difficult to compare findings from various studies due to different sampling methods and considerable year-to-year variability. Nevertheless, these studies clearly indicate a wide geographical distribution and show that most detections occur between July and September and mainly concern the genotypes Europe 2, Europe 3 and Africa 3.

Following the 2018 avian epidemic in the Netherlands, in a retrospective study of blood donors, seven of 12,040 (0.06%) donations were positive for USUV-RNA, blood from three of them were transfused to three immunocompetent recipients who did not develop infection [39]. To date, no transfusion-transmitted infections of USUV have been reported, but it should be noted that these may be undetected given the mostly asymptomatic nature of USUV infections and the lack of routine screening for USUV of recipients.

Currently, the French public health authorities do not consider the risk of USUV transmission by transfusion as major and do not recommend any specific measures to prevent blood transmission [40].

The increasing number of human cases of WNV infection and a geographical expansion of the affected areas justified in 2015, 2017, 2018, 2019 and 2022 the implementation of the WNV-NAT screening for blood donations. In 2023, for the first time, autochthonous cases of WNV infection were reported on the Atlantic coast (Gironde (D-33) and Charente (D-16)), leading to a further extension of the NAT screening programme, which resulted in the present study reporting both WNV and USUV infections.

Infections with USUV in our study were detected over 14 weeks (mid-July to mid-October 2023) with a wide geographical distribution. In contrast, cases with WNV RNA were limited to the Mediterranean region (n = 1) and to the new emergence zone (n = 4) on the Atlantic coast during a shorter period of 5 weeks (mid-August to mid-September).

These findings suggest varying eco-epidemiology of WNV and USUV, despite similarities in primarily affecting avian populations and being transmitted by mosquitoes of the *Culex* genus. However, in the absence of systematic detection of these viruses across the whole country and throughout the entire period of the vector activity, the current data should be treated with caution and complemented by studies in vectors, birds and Equidae. Nevertheless, since 2023, USUV and WNV appear to have become more geographically widespread, with a notable extension towards the north of the country. In the summer of 2025, WNV virus was detected in the Paris region [41]. Combined with a long seasonal detection period, such as that observed among blood donors (14 weeks — a figure that is certainly an underestimate given the circulation of the virus within the avian population), this could indicate endemic establishment.

Therefore, better characterisation of the avian and entomological ecoepidemiology of WNV and USUV is needed to understand the determinants of the viral circulation (and co-circulation), including (i) endemic maintenance and reintroductions by migratory bird populations; (ii) immunity in avian infections; (iii) susceptibility of different species to infection and clinical expression, and (iv) determinants for vector competence.

Distinguishing between WNV and USUV by specific RT-qPCR after a positive molecular screening test is challenging, particularly when viral loads are low. The NAT screening assay is considered more sensitive than the species-specific confirmatory RT-qPCR assays. However, when differentiation by RT-qPCR is not possible, a sample collected a few weeks after donation can be used to measure seroconversion and/or changes in neutralising antibody titres. But serology has also limitations: cross-reactivity between Orthoflaviviruses, particularly between USUV and WNV, and the lack of a commercial kit for anti-USUV IgM. Ultimately, species-specific, highly sensitive RT-qPCR protocols would provide the most reliable information, following screening by NAT.

## Conclusion

This study confirms the circulation of USUV and human exposure to USUV in mainland France, which supports the findings from other European countries. Although the prevalence of USUV and WNV infections among blood donors was low, detection of these viruses may become more frequent. We encourage development of highly sensitive tests capable of distinguishing WNV from USUV as well as the conducting seroprevalence studies to provide more accurate information on exposure to this virus and its geographical distribution. A One Health approach is necessary to understand the determinants of USUV circulation, the clinical consequences of infection in humans and to improve the safety of substances of human origin.

## Data Availability

The data that support the findings of this study are available in an aggregated and anonymised format on reasonable request from the corresponding author. Sequence and analysis files are available from https://github.com/rklitting/WNV_USUV_EFS_2023.

## Supplementary Data

223000 Supplementary Material
