# Supplementary material for: Identification of Usutu virus in blood donors as part of molecular screening for West Nile virus, France, 2023
Source: Euro Surveill. 2026 Oct 29;31(43):2600044. doi: 10.2807/1560-7917.ES.2026.31.43.2600044 (PMC13722931; doi:10.2807/1560-7917.ES.2026.31.43.2600044)
Supplement: Supplementary file 1 [file 26-00044_GALLIAN_Supplement.pdf]

Supplementary material

"This supplementary material is hosted by *Eurosurveillance* as supporting information alongside the article [Identification of Usutu virus in blood donors as part of molecular screening for West Nile virus, France, 2023], on behalf of the authors, who remain responsible for the accuracy and appropriateness of the content. The same standards for ethics, copyright, attributions and permissions as for the article apply. Supplements are not edited by *Eurosurveillance* and the journal is not responsible for the maintenance of any links or email addresses provided therein."

Supplementary Table S1 : USUV molecular detection in blood donor population, Europe, 2016-2024

Country	Period considered	Detected cases	Rate	Genotype (number)	Method (Pool size)	Ref (PMID)
Germany (Aachen)	September 2016	1	1/13023	Eu3	Cobas taqman (P16)	Cadar et al.<br>(28422005)
Austria	July-August 2017<br>Summer 2016	6<br>1	6/12047<br>1/70864	Eu2 (5)	Cobas 8800 (P19)	Bakonyi et al.<br>(29043962)
Italy<br>(Lombardy)	2016-2018	25	25/73964	NS*	Cobas 6800 (NS*)	Percivalle et al.<br>(32013152)
Italy<br>(Lazio/Lombardy)	August-September 2017-2018	9	9/ NS*	Eu2 (5)<br>Eu3 (1)<br>Eu4 (2)	Cobas (NS*)	Carletti et al.<br>(30670139)
Austria	June-September 2018	18	18/31598	Eu2 (14)<br>Afr3 (2)	Cobas 8800 (NS*)	Aberle et al.<br>(30376913)
Netherland<br>(retrospective)	August 2018<br>September 2018	6<br>1	6/4416<br>1/4936	Eu3 (2)	Cobas 6800 USUV home test<br>(P8)	Zaaijer et al.<br>(31270821)
Germany	2020-2023	10	10/ 26,4 millions	Eu2 (3)<br>Eu3 (7)	Several methods<br>Mini-pools (1 to 96)	Orru et al.<br>(40017391)
Spain<br>(Balearic Islands/Catalonia)	July-September 2024	3	3/95357	Afr3 (2)	Cobas / Panther<br>(individual)	Jimenez et al.<br>(41186283)
Austria	2024	27	27/ NS*	Eu2 (14)<br>Afr3 (1)	Cobas (NS*)	Florian et al.<br>(41508892)

* : Not Specified

Genotypes : Eu2, 3, 4 = Europe 2, 3, 4 ; Afr3 = Africa 3
